# A methodological consideration for blood lead concentrations obtained from the earlobe in Japanese adults occupationally unexposed to lead

**DOI:** 10.1186/s12199-017-0685-9

**Published:** 2017-12-11

**Authors:** Nozomi Tatsuta, Kunihiko Nakai, Miyuki Iwai-Shimada, Futoshi Mizutani, Katsuyuki Murata, Yoichi Chisaki, Hiroshi Satoh

**Affiliations:** 1 Development and Environmental Medicine, Tohoku University Graduate School of Medicine, Sendai, Japan; 2 Centre for Health and Environmental Risk Research, National Institute for Environmental Studies, Tsukuba, Japan; 3 Institute of Environmental Ecology, IDEA Consultants, Inc., Yaizu, Japan; 4 Department of Environmental Health Sciences, Akita University Graduate School of Medicine, Akita, Japan; 5 Environmental Health Science, Tohoku University Graduate School of Medicine, Sendai, Japan; 6 2-1 Seiryo-machi, Aoba-ku, Sendai, Miyagi 980-8575 Japan

**Keywords:** Lead, Capillary blood collection, Earlobe

## Abstract

**Background:**

Neuropsychological effects of considerably low levels of lead exposure are observed in children, and a reliable and possibly painless technique that can detect such levels is required for the assessment of such exposure. We examined whether the blood lead (BPb) concentrations obtained from the earlobe were as valid and useful as those from the median cubital vein.

**Methods:**

Paired blood samples were collected from the earlobe and cubital vein of 112 Japanese participants occupationally unexposed to lead, and the BPb levels were determined using ICP-MS.

**Results:**

The limit of detection of BPb for the ICP-MS method was 0.015 μg/dL, and there was no participant with a BPb level below this limit. The median values of BPb concentrations were 0.91 (range, 0.41–2.48) μg/dL for earlobe blood using a 175-μL capillary tube and 0.85 (0.35–2.39) μg/dL for venous blood using a 5-mL vacuum tube. There was a significant correlation between the earlobe BPb levels and cubital vein BPb levels (Spearman rank correlation *r*_S_ = 0.941), though the earlobe BPb levels were significantly higher than the cubital vein BPb levels. Most of the participants regarded earlobe puncture as a painless method.

**Conclusions:**

These data suggest that earlobe BPb levels can be used to assess lead exposure in children. Blood collection using a capillary tube should be done carefully and promptly because slow withdrawal may lead to measurement bias.

## Background

Children are more vulnerable to lead toxicity than adults [1, 2]. For this reason, most epidemiological studies on the health effects of lead have focused on children. Recent reports demonstrated the impact of postnatal exposure to lead at levels of less than 5 μg/dL of blood on children’s intelligence [3–5]. Furthermore, the Faroese birth cohort study found cognitive deficits due to prenatal lead exposure in 7- and 14-year-old children [6], whose average cord-blood concentration was 1.6 μg/dL (interquartile range, 1.2–2.2 μg/dL). Thus, since neuropsychological effects of lead exposure at considerably low levels are observed in children, a reliable and simple technique that can detect such levels of lead is required for risk assessment of lead.

The LeadCare Plus (ESA Biosciences, Inc., Chelmsford, MA, USA) has been used as a point-of-care testing device for analyzing whole blood lead (BPb) samples [7]. It needs only 50 μL (approximately 2 drops) of whole blood obtained from the fingertip using a capillary tube, to measure the exposure level of lead. According to the manufacturer’s instructions [8], the detection limit is between 1.9 and 65 μg/dL. On the other hand, the geometric mean of BPb levels in Japanese children was found to be around 1.0 μg/dL [9, 10], indicating that the LeadCare Plus would be useless for Japanese children. In addition, Japanese infants and children tend to fear injection needle. Since subjects aged 22 to 94 (mean 74.4) years felt a significantly lower level of pain when the earlobe rather than fingertip was pricked [11], therefore, it would be meaningful to establish an acceptable method for BPb determination by using blood obtained from the earlobe, instead of the fingertip. This method would be favorable for application to children because earlobe puncture is less visible compared to fingertip puncture. In this study, we collected blood samples from the earlobe and median cubital vein and compared their BPb levels to clarify whether lead concentrations of blood from the former were as valid and useful as those from the latter.

## Methods

### Study subjects

Participants were recruited, and blood samples were collected from the earlobe and median cubital vein. We explained the nature of the procedures used in the present study to Japanese subjects aged 20 years and over living in Miyagi and Shizuoka prefectures in Japan, and 112 participants provided written informed consent. The exclusion criterion for this study was present or previous exposure to lead occupationally. This protocol was approved by the Medical Ethics Committee of the Tohoku University Graduate School of Medicine.

### Contamination check

To avoid lead contamination through sampling devices during earlobe blood sampling, we used blood collection tubes with the lowest lead level. We filled three kinds of heparinized glass capillary tubes, thereafter sealed with caps, with physiological saline solution or 1/100-diluted HNO_3_ and left them standing overnight at 4 °C. After that, we measured the lead levels in the solutions recovered from these tubes as described below; the results are shown in Table 1. Based on this, a 175-μL capillary tube (J473763, Siemens Healthcare Diagnostics Manufacturing Ltd.) was selected.

**Table 1:** Lead levels in solutions recovered from capillary tubes: analysis of lead contamination

	Lead levels (μg/dL)	
Heparinized glass capillary tube	Physiological saline solution	1/100-diluted HNO_3_
TERUMO Corp. (150 μL, cap, VC-C110HL)	0.429	0.801
Thermo Fisher Scientific Inc. (250 μL, KN3131665)	0.119	0.136
Siemens Healthcare Diagnostics Manufacturing Ltd. (175 μL, cap, J473763)	0.015	0.019

### Blood collection and BPb analysis

Nurses put on gloves, washed their hands with foam soap, and wore personal protective equipment during blood collection. Before blood collection, the arm was swabbed with alcohol. A venous blood sample was drawn from the cubital vein into a 5-mL vacuum tube (Venoject II VP-P050K, TERUMO Corp., Tokyo, Japan) pre-treated with ethylenediaminetetraacetic acid disodium (EDTA-2Na) anticoagulant using a 23-gauge needle (TERUMO Corp., Tokyo, Japan), and about 3 mL of venous blood was collected. Following venous blood sampling, earlobe blood collection was performed. An alcohol swab was used to remove dust. Puncture of the inferior border of the earlobe was done using a disposable 1.8-mm contact-activated sterile lancet (Safety Lancet BD Microtainer, Tokyo, Japan), and blood was collected directly into a 175-μL heparinized glass capillary tube (i.e., J473763, Siemens Healthcare Diagnostics Manufacturing Ltd.) and treated in a similar procedure of the contamination check. The paired samples were transported under cool conditions to a laboratory in Shizuoka prefecture where the capillary tube samples were immediately analyzed, and vacuum tube samples were stored at − 80 °C until analysis. To recover earlobe blood from the capillary tube, we (1) removed the bottom cap from the capillary tube that was filled with the blood sample, (2) placed a pre-weighed blank vial under the capillary tube, (3) replaced the upper cap of the tube and put the tip of micropipette on the top of the tube, (4) pushed air into the capillary tube using a micropipette, until all the blood was forced out into the vial, (5) repeated this until all of the blood in tube was transferred to the vial, and (6) weighed the vial with the blood sample and deducted the mass of the blank vial. In this way, we could calculate the mass of the blood sample.

BPb concentrations of the paired samples were determined by inductively coupled plasma mass spectrometry (Agilent 7900 ICP-MS; Agilent Technologies Japan, Tokyo, Japan) using thallium (Wako Pure Chemical Industries, Ltd., Osaka, Japan) as the internal standard. Whole blood was diluted 1:20 with an alkaline diluent containing 2% butanol (RoHS compliant; Wako Pure Chemical Industries Ltd., Osaka, Japan), 0.05% polyoxyethylene(10) octylphenyl ether (Practical grade; Wako Pure Chemical Industries, Ltd., Osaka, Japan), 0.05% EDTA (Dojindo Laboratories, Kumamoto, Japan), and 0.1% tetramethylammonium hydroxide (Super special grade; Wako Pure Chemical Industries, Ltd., Osaka, Japan). The standard for lead was purchased from Wako Pure Chemical Industries (Osaka, Japan). Milli-Q water used in the experiment (> 18.2 MΩ) was deionized and purified using a Milli-Q system (Merck KGaA, Darmstadt, Germany). Method detection limit (MDL) for lead analysis, calculated using the method described by Currie [12], was 0.015 μg/dL.

### Statistical analysis

Spearman rank correlation coefficients (*r*_S_) were calculated to determine the relationship between BPb levels in the earlobe and median cubital vein samples. The Wilcoxon signed rank test was used to compare BPb levels in the paired earlobe and median cubital vein samples. Sex differences in basal characteristics were analyzed by the Student *t* test, Fisher exact test, and Mann-Whitney *U* test. All analyses, with two-sided *p* values, were performed using SPSS Ver. 23.0 (SPSS Japan, Tokyo), and the significance level was set at 5%.

## Results

A preliminary examination was conducted using one subject to confirm the practicality of our procedures (Table 2). Some blood samples were transferred directly to the laboratory in Shizuoka and others were transferred there via a courier delivery service. The BPb levels seemed not to differ between blood samples collected from the earlobe and the median cubital vein. In addition, the duration between blood collection and BPb analysis, i.e., within 5 days, hardly affected the BPb levels. As a result, all blood samples were transported to the laboratory by courier delivery.

**Table 2:** Preliminary examination of blood lead (BPb) levels in one subject

	Lead (μg/dL)^a^
Blood samples transferred to the laboratory directly	
BPb levels by different blood collection methods	
Measurement vial blood collection from the right earlobe	0.84
Capillary blood collection from the right earlobe	0.87
Capillary blood collection from the left earlobe	0.91
Vacuum tube blood collection from the median cubital vein	0.83
BPb levels analyzed after storing blood obtained from the median cubital vein in capillary tubes^b^	
1-day storage	0.84
2-day storage	0.84
3-day storage	0.85
5-day storage	0.84
BPb levels in blood samples transferred to the laboratory via a courier delivery at 4 °C.	0.89

^a^Average value of two measurements for lead levels in blood collected in a measurement vial, capillary tube or vacuum tube

^b^Capillary tube samples were stored at 4 °C until analysis

Analytical quality assurance includes analyses of method blanks, duplicates, and the reference material. Method blanks did not exceed the MDL of our ICP-MS measurement protocol. Duplicates for about 10% of all samples (*n* = 10) were evaluated for repeatability, and the difference of each duplicate was less than 5%. Regarding the precision of the analytical measurements, the measured value of blood reference material for lead (Seronorm™ Trace Elements Whole Blood L-1, Lot 1406263) was 1.06 μg/dL and within the acceptable range (0.79 to 1.19 μg/dL) of the reference value (0.99 μg/dL). The precision was assessed by measuring the reference material two times on three different days, and the relative standard deviation (RSD) was 1.1%.

**Fig. 1:**
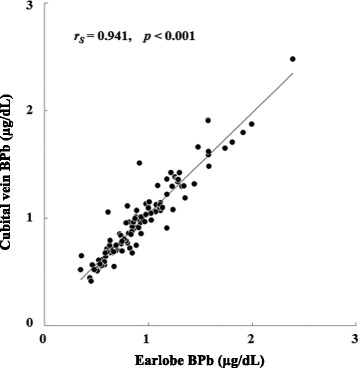
Relationship between blood lead (BPb) concentrations measured in samples obtained from the earlobe and the median cubital vein from 112 subjects: *r*_S_ indicates the Spearman rank correlation coefficient. The linear regression equation is as follows: [cubital vein BPb] = 0.959 × [earlobe BPb] + 0.005

After the above confirmation, the two types of blood samples were collected from each participant. The mean age of the participants was 32 (range, 20–66) years. There was no participant with a BPb level below the MDL, and the BPb levels from the earlobe and the cubital vein ranged from 0.41 to 2.48 (median 0.91) μg/dL and 0.35 to 2.39 (median 0.85) μg/dL, respectively. The BPb levels from the earlobe were significantly higher than those in samples collected from the cubital vein (*p* < 0.001), but earlobe BPb levels significantly correlated with cubital vein BPb levels as shown in Fig. 1. Table 3 presents the basal characteristics and BPb levels of the 62 male and 50 female participants. Although age did not significantly differ between the sexes, earlobe and cubital vein BPb levels in males were significantly higher than those in females. Further, earlobe BPb levels were significantly higher than cubital vein BPb levels both in the males and females (*p* = 0.005 and 0.003, respectively). The cubital vein BPb levels significantly correlated with age (*r*_S_ = 0.240), but the correlation coefficient of the earlobe BPb (*r*_S_ = 0.174) was not statistically significant. The proportion of smokers and ex-smokers was 20.5%, and both earlobe and cubital vein BPb levels were significantly higher in the smoker/ex-smoker group (median 1.10 and 1.14 μg/dL, respectively) than in the nonsmoker group (0.85 and 0.82 μg/dL), but the significance disappeared after adjusting for age (*p* > 0.1).

**Table 3:** Age (mean ± SD), smoking status (number and % in parenthesis), and blood lead (BPb) concentrations (median) of 112 participants

	62 males	50 females	*p* value^a^
Age at examination (years)	34 ± 13	31 ± 11	0.183
Smokers + ex-smokers (%)	17 (27.4)	6 (12.0)	0.060
Earlobe BPb level (μg/dL)	0.99	0.73	< 0.001
Cubital vein BPb level (μg/dL)	0.93	0.74	< 0.001

^a^Student *t* test, Fisher exact test, and Mann-Whitney *U* test were done for age, smoking status, and BPb concentrations, respectively

## Discussion

This study aimed to confirm the acceptability and accuracy of BPb concentrations from the earlobe. Currently, the MDL of BPb analysis using the IPC-MS (e.g., 0.015 μg/dL in this study) is dropping, thanks to innovation; for this reason, lower levels of BPb, for instance, 1.07 μg/dL (geometric mean) in 352 children aged 6.6 ± 3.8 years [9] and 0.96 μg/dL in 229 children aged 9–10 years [10], can be determined. The accuracy and precision of BPb analysis using the reference material of lead were indicated to be within the acceptable range and a low RSD (1.1%). In addition, most of the participants, not including children, felt that blood sampling from the earlobe was painless in comparison with that from the cubital vein. Therefore, this blood sampling method appears to be suitable for children.

Concerning the accuracy of this method, the earlobe BPb showed a strong relation to the cubital vein BPb (*r*_S_ = 0.941) as illustrated in Fig. 1, but the former was approximately 4% higher than the latter. On the other hand, previous studies reported 10–30% higher BPb levels in capillary blood obtained by finger than by venous puncture [13, 14], implying a large deviation as compared to that from the earlobe BPb levels. One possible reason for the slightly high earlobe BPb level could be that blood sampling from the earlobe was done using a 175-μL capillary tube; that is, since its collected volume was extremely less than that of the 5-mL vacuum tube for the cubital vein, it is likely that a minute volume of water in the blood collected from the earlobe evaporated due to its air and skin contact during sampling or might have adhered to the capillary tube, though we did not examine hematocrit in blood from the earlobe and cubital vein. For this reason, a slow or botched procedure for blood sampling and too small a volume of blood might result in measurement bias, i.e., overestimation of the BPb level. Another possible reason is that different reagents (i.e., heparin and EDTA) were used to keep the blood from hardening.

In the present study, earlobe and cubital vein BPb levels were higher in the males than in the females and the latter BPb showed a weak but significant correlation with age. In Japan, since leaded vehicle fuel was used until 1975, the participants older than 45 years may have been exposed to lead environmentally. In fact, the atmospheric lead concentrations in central, suburban, and background Tokyo were approximately 1.7 μg/m^3^ in 1969 and have been below 0.2 μg/m^3^ since 1978 [15]. As a result, mean BPb levels in Japanese men occupationally unexposed to lead were 10.3 (range, 5.5–15.7) μg/dL in 1983 [16], 3.9 (range, 1.8–6.9) μg/dL in 1995 [17], and 5.5 ± 2.5 (SD) μg/dL in 1998 [18], and geometric means of BPb in Japanese women ranged from 2.1 to 6.2 (median 3.3) μg/dL in 1980, 1.5 to 3.8 (median 2.5) μg/dL in 1990, and 1.7 to 2.2 (median 1.9) μg/dL in 1991–1998 [19], indicating that BPb levels decreased each year; whereas, the analytical methods for lead may have differed among these studies. Thus, BPb level appears to increase with aging because of their exposure to relatively high environmental lead levels in the past [20] and the half-life for lead in the bone (about 27 years) is considerably longer than that in the blood (about 28–36 days) [21], and BPb levels are generally higher in males than in females, inasmuch as the major sources of lead are house dust and diet [19–23], and males consume more food than females.

In our study, smokers and ex-smokers had higher BPb levels than nonsmokers; but, since the significant difference disappeared after adjusting for age, the effect of smoking would be limited, rather due to the fact that the smokers and ex-smokers were older than the nonsmokers (45 ± 12 and 29 ± 9 years old, respectively). On the other hand, the mean content of lead in filter-tipped cigarettes produced between 1960 and 1980 was 2.4 μg/g, and approximately 5% of this lead was estimated to be inhaled [21]. In addition, Kaji and coworkers [24] reported that children whose parents smoked in the same room had significantly higher BPb levels than those of nonsmoking parents. Thus, the effects of smoking on the BPb concentration should not be treated lightly.

## Conclusion

The earlobe BPb levels strongly correlated with cubital vein BPb levels (*r*_S_ = 0.941), and the procedure seems to be generally painless. Therefore, this method may be applicable to assess BPb level in children, regarding its accuracy and acceptability, whereas the BPb from the earlobe needs the following adjustment (e.g., [BPb from the cubital vein] = [BPb from the earlobe] × 0.96). In any case, blood sampling with a capillary tube should be done carefully and promptly because a slow pace and a too small volume of blood may lead to measurement bias.

## Abbreviations

BPb: Blood lead
ICP-MS: Inductively coupled plasma mass spectrometry
MDL: Method detection limit
RSD: Relative standard deviation
SD: Standard deviation
